# Complete mitochondrial DNA sequence of the *Psammocora profundacella* (Scleractinia, Psammocoridae): mitogenome characterisation and phylogenetic implications

**DOI:** 10.3897/BDJ.9.e62395

**Published:** 2021-04-19

**Authors:** Peng Tian, Jiaguang Xiao, Zhiyu Jia, Feng Guo, Xiaolei Wang, Wei Wang, Jianjia Wang, Dingyong Huang, Wentao Niu

**Affiliations:** 1 Third Institute of Oceanography, Ministry of Natural Resources, Xiamen, China Third Institute of Oceanography, Ministry of Natural Resources Xiamen China

**Keywords:** evolutionary relationships, mitochondrial genome, next-generation sequence (NGS), phylogenetics

## Abstract

Complete mitochondrial DNA sequence data have played a significant role in phylogenetic and evolutionary studies of scleractinian corals. In this study, the complete mitogenome of *Psammocora profundacella* Gardiner, 1898, collected from Guangdong Province, China, was sequenced by next-generation sequencing for the first time. *Psammocora profundacella* is the first species for which a mitogenome has been sequenced in the family Psammocoridae. The length of its assembled mitogenome sequence was 16,274 bp, including 13 protein-coding genes, two tRNAs and two rRNAs. Its gene content and gene order were consistent with the other Scleractinia species. All genes were encoded on the H strand and the GC content of the mitochondrial genome was 30.49%. Gene content and order were consistent with the other Scleractinia species. Based on 13 protein-coding genes, Maximum Likelihood phylogenetic analysis showed that *P. profundacella* belongs to the “Robust” clade. Mitochondrial genome data provide important molecular information for understanding the phylogeny of stony corals. More variable markers and additional species should be sequenced to confirm the evolutionary relationships of Scleractinia in the future.

## Introduction

Scleractinia (Cnidaria, Anthozoa), including numerous hermatypic corals, have always been highlighted by ecologists and taxonomists for their important role in maintaining the balance of ecosystems in shallow tropical and subtropical seas (Fukami et al. 2000). Traditionally, reef-building corals were described, using morphological characteristics of their skeletons, whereas the skeletal structures, especially of colonial corals, can be extremely variable due to environment-induced phenotypic plasticity. In addition, it was difficult to discriminate cryptic species and species that have arisen via introgression or hybridisation without genetic and ecological data (Niu et al. 2018,Combosch and Vollmer 2015, Richards and Hobbs 2015).

Molecular technologies have changed the taxonomic landscape and the integration of morphological and molecular analyses have promoted a more rigorous classification and precise phylogeny of stony corals (Kitahara et al. 2016). Though mitochondrial genes have been known for producing erroneous phylogenetic inferences amongst anthozoans, mitochondrial phylogenies of scleractinians may still provide insight into mitochondrial and gene evolution and may provide preliminary insights into evolutionary relationships (Lin et al. 2014).

Cnidarian mitogenome data contain important phylogenetic information for understanding its evolutionary history (Kayal et al. 2013). Single or multiple gene analysis of mitochondrial genes have been used to infer phylogenetic relationships amongst scleractinians (Arrigoni et al. 2020, Kitahara et al. 2016). Data of complete mitochondrial genomes have also become important sources for assessing scleractinian phylogenies due to the declining cost of next-generation sequencing (NGS) technologies (Jex et al. 2010, Niu et al. 2018, Schuster 2008). Nevertheless, there are more than 1600 species, whereas only approximately 100 complete mitogenomes of Scleractinia species have been collected in NCBI (https://www.ncbi.nlm. nih.gov/) to date (Hoeksema and Cairns 2020).

*Psammocora profundacella* Gardiner, 1898 is a species of small-polyp stony coral with grey, brown, tan or cream colours, belonging to the family Psammocoridae (Chevalier and Beauvais 1987). Its colonies are sub-massive or encrusting, corallites are in short valleys and walls are rounded, although they may have a central ridge. Petaloid septa and enclosed petaloid septa are always present and rice-grain shaped (Benzoni et al. 2010, Benzoni et al. 2007). There is only one genus, *Psammocora* Dana 1846, in the Psammocoridae and is widely distributed in the Indo-Pacific. It had been placed in the family Siderastreidae until Benzoni et al. (2007) published strong evidence to distinguish *Psammocora* from other siderastreids and resurrected Psammocoridae to accommodate this genus. Benzoni et al. (2010) revised all 24 species in *Psammocora*, reducing the number to seven species using a combination of morphological and molecular analyses; subsequently, Randall (2015) identified a new species, *Psammocora eldredgei*.

In the present research, the complete mitochondrial genome of *P. profundacella* was sequenced using NGS and its genome structure was analysed for the first time. Simultaneously, it was also the first species within the family Psammocoridae for which the mitogenome had been sequenced. The phylogenetic analyses of *P. profundacella*, based on protein coding genes (PCGs) of the mitogenome, combined with 81 other scleractinians, will help determine its taxonomic status and facilitate further study on stony coral evolutionary and phylogenetic relationships.

## Material and methods

### Sample collection and genomic DNA extraction

**Figure 1.**
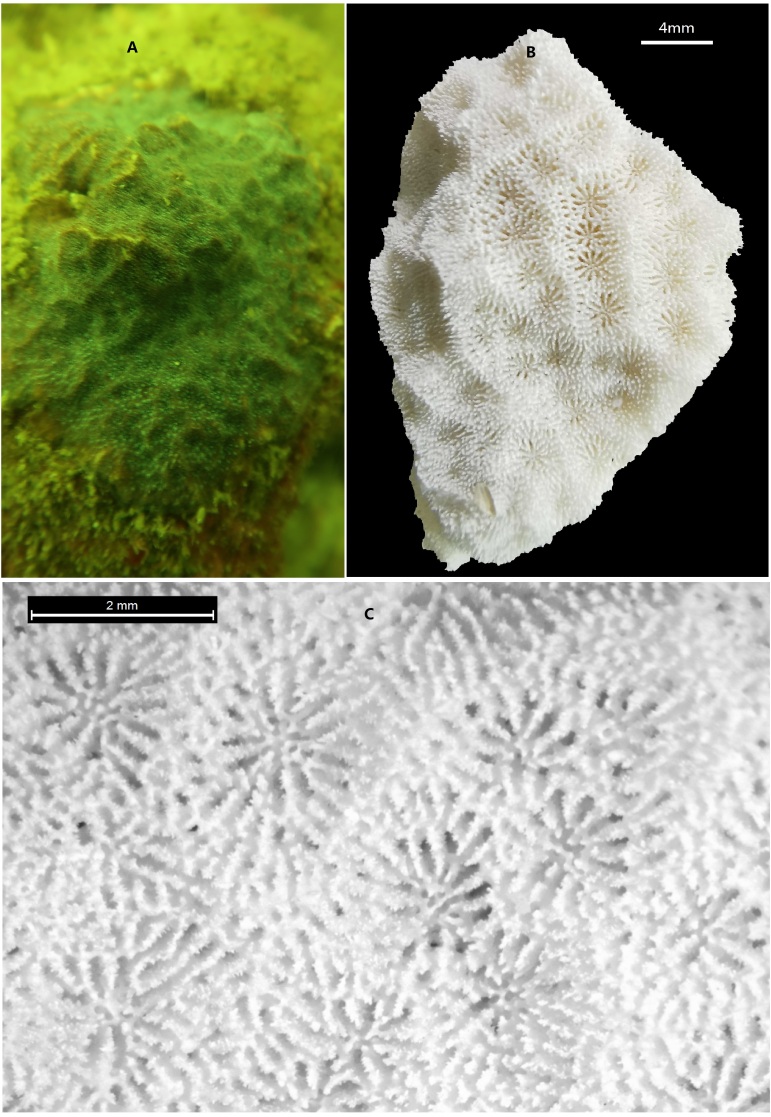
Photos of *P. profundacella* in this study. (A) In-situ photograph of *P. profundacella*; (B) Skeleton photograph of *P. profundacella*; (C) Microskeletal photograph of *P. profundacella*.

A specimen of *P. profundacella* (Fig. 1) was collected from Yangmeikeng of Daya Bay in Guangdong Province and it was kept in our Coral Sample Repository with a special code, 20190718-D17. Identification was conducted according to the description of Benzoni et al. (2010): calices are between 1.4 and 1.7 mm in diameter, the columella is made of one central process surrounded by 4–6 granules positioned at the inner end of the septa and up to 6 septa reaching the fossa are petaloid. Complete genomic DNA was extracted using the DNeasy Blood and Tissue Kit (Qiagen, Shanghai, China). Electrophoresis with 1% agarose gel was used to measure the integrity of the genomic DNA and the spectrophotometer NanoDrop 2000 (Thermo Scientific, USA) was used to measure the genomic DNA concentration.

### Genome sequence assembly and analyses

After DNA extraction and quality detection, the sequencing library was produced using the Illumina Truseq™ DNA Sample Preparation Kit (Illumina, San Diego, USA) according to the manufacturer's recommendations. Five µg of double-stranded DNA was sheared to ~ 300 bp using a M200 Focused-ultrasonicator (Covaris, Woburn, MA, USA). The prepared library was loaded on the Illumina HiSeq X Ten platform for PE 2 × 150 bp sequencing. The quality and quantity of data were assessed by FastQC (Andrews 2010). Filtered clean reads were obtained by removing reads containing poly-N regions, adapters and by eliminating low-quality reads using fastp (Chen et al. 2018). The median of insert sizes and average read length were used to reconstruct the mitochondrial genome via NOVOPlasty 3.8.3 (Nicolas et al. 2016). A total of 60,910 of 128,526,740 raw reads (approximately 0.05%) were de novo assembled to produce the mitogenome with the guidance of seed sequence and the average coverage was 565×.

### Mitogenome analyses

The circularised contig was submitted to MITOS (Bernt et al. 2013) WebServer (http://mitos.bioinf.uni-leipzig.de/index.py) for preliminary mitochondrial genome annotation. We then identified and annotated all 13 PCGs and two rRNA genes by alignments of homologous mitogenomes with other scleractinians that had been reported through BLAST searches in NCBI. All the PCG codon usage and nucleotide frequencies were obtained through Molecular Evolutionary Genetics Analysis software MEGA7 (Kumar et al. 2016). tRNA genes were identified by comparing the results predicted by tRNAscan-SE 2.0, based on its unique cloverleaf secondary structure information and we also validated the result using ARWEN (Laslett and Canbäck 2008, Lowe and Chan 2016).

### Phylogenetic analyses

The phylogenetic position of *P. profundacella* was inferred using 13 tandem mitogenome PCG sequences (ND5 + ND1 + Cytb + ND2 + ND6 + ATP6 + ND4 + COIII + COII + ND4L + ND3 + ATP8 + COI) together with 81 other Scleractinia species that we obtained from GenBank (see Suppl. material 1). We used MEGA7 to choose the best-fitting model, based on the Akaike Information Criterion (AIC) and then constructed a Maximum Likelihood (ML) tree with 500 bootstrap replicates.

## Results and Discussion

### Characteristics and composition of mitogenome

**Figure 2.**
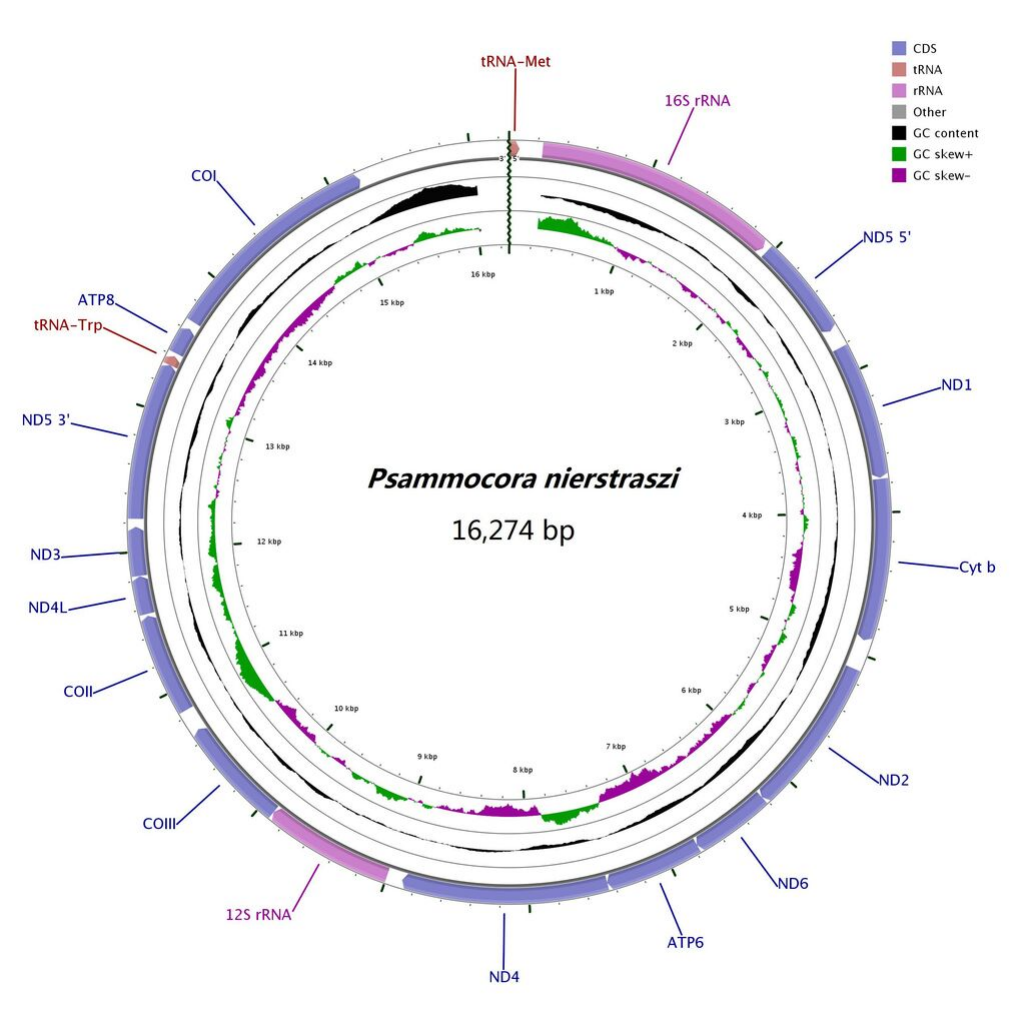
The mitochondrial genome of *P. profundacella*. Gene order and positions are shown. COI, COII and COIII refer to the cytochrome oxidase subunits, Cyt *b* refers to cytochrome b and ND1-ND6 refers to NADH dehydrogenase components. All the genes are encoded on the H-strand.

**Figure 3.**
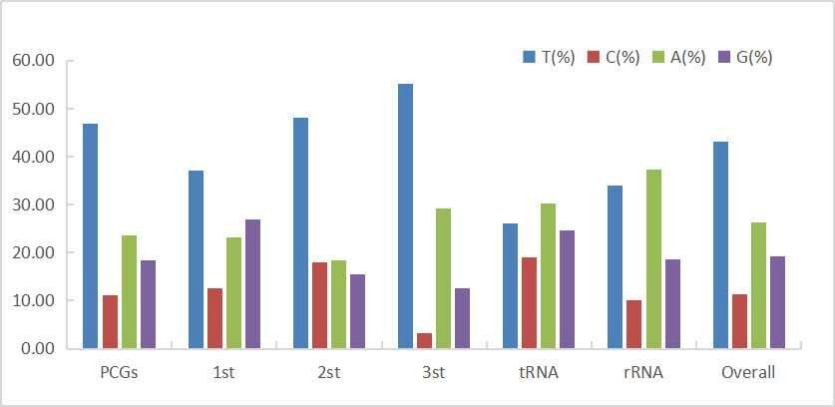
Codon usage bias in different regions of mitochondrial genome of *P. profundacella*.

The mitochondrial genome size of *P. profundacella* (GenBank accession number: MT576637) was 16,274 bp, including 13 PCGs, 2 tRNA (tRNA^Met^, tRNA^Trp^) and 2 rRNA genes (see Table 1,Fig. 2). The mitogenome of *P. profundacella* offered no distinct structure and its gene order, gene identity and gene number were the same as those of published stony coral mitogenomes (Wang et al. 2013). The base composition of complete mitogenome was 26.34% A, 11.32% C, 19.17 G and 43.17% T, which showed a higher AT content (69.51%) than GC content (30.49%) (see Fig. 3, Table 2). In addition, all genes remained encoded on the H-strand.

### Protein-coding genes and its codon usages

**Figure 4.**
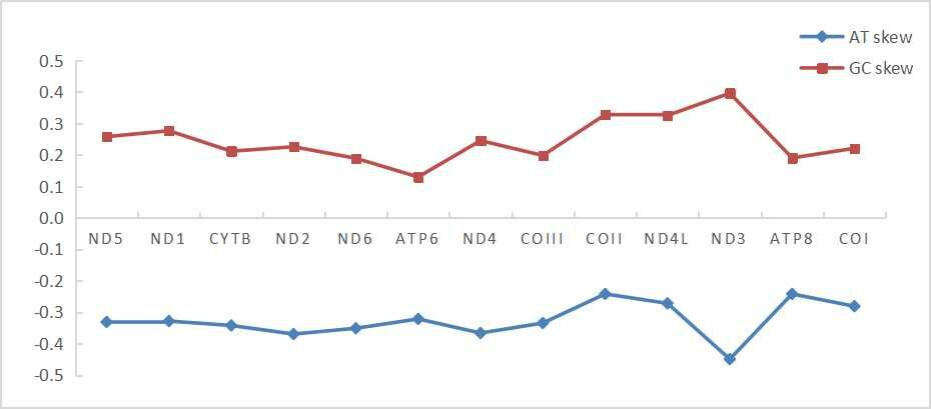
The PCGs’ AT-skew and GC-skew of mitochondrial genome of *P. profundacella*.

**Figure 5.**
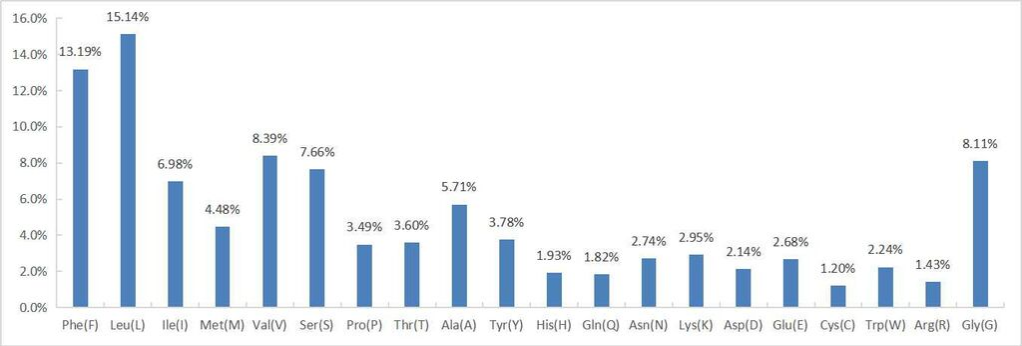
The PCGs’ codons use frequency of mitochondrial genome of *P. profundacella*.

The length of all 13 protein-coding genes sequence was 11,511 bp, with base composition of 23.6%, 11.2%, 18.4% and 46.8% for A, C, G and T, respectively. ND5 gene had an intron insertion of 9,549 bp. The start codon of all PCGs used ATG, except for Cyt *b* using TTA and ND2 using ATT. Three PCGs (COII, ND4 and ND5) terminated with TAG, the other ten PCGs (ATP6, Cyt *b*, ND1, ND2, ND3, ND4L, ND6, ATP8, COII and COIII) stopping with TAA. The shortest gene was ATP8 (195 bp) and the longest gene was ND5 (1,812 bp). The intergenic region between cytb and ND2 was 208 bp (see Table 1). According to the results of AT-skew and GC-skew analysis (Fig. 4), all PCGs showed a stronger nucleotide asymmetry, with AT-skew higher than GC-skew. Amongst L, F, V, G and S, codon use frequency was higher, accounting for 52.5% of a total of 3837 codons. Amongst the 20 amino acids, the majority were were non-polar amino acids which accounted for 68.0%; the minority were polarity-charged amino acids accounting for 11.1% and the remainder were polar amino acids which accounted for 20.5% (Fig. 5).

### rRNA and tRNA genes

**Figure 6.**
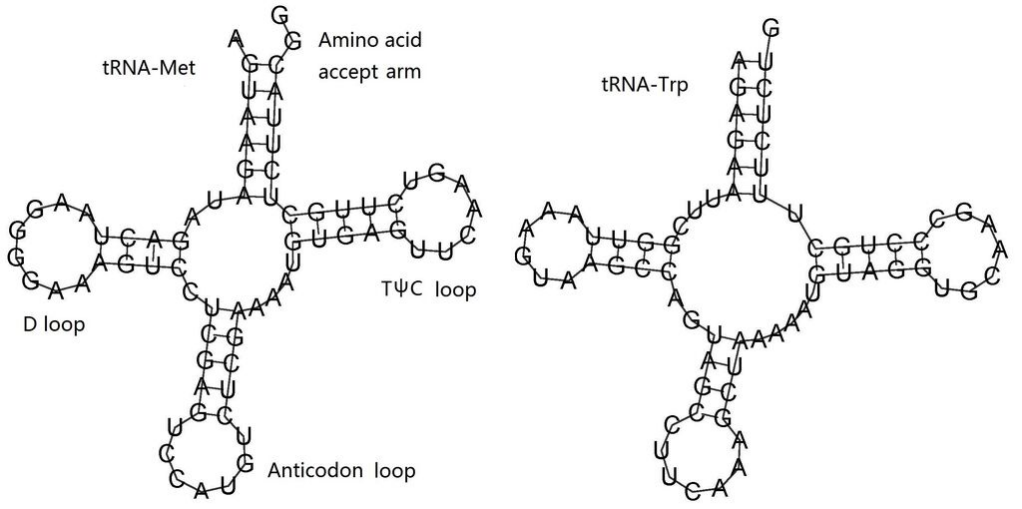
Putative secondary structures of two tRNA of *P. profundacella*.

The encoding genes 12S and 16S rRNA in *P. profundacella* were 906 bp and 1,704 bp in size, respectively. Both the two rRNAs’ base composition was 37.32% A, 10.15% C, 18.54% G and 33.98% T. There were also two tRNA encoding genes- tRNA^Met^ (72 bp) and tRNA^Trp^ (70 bp). They were folded into the classic cloverleaf structure which included an amino acid accept arm, DHU loop, anticodon loop and TψC loop (Fig. 6).

### Phylogenetic analyses

**Figure 7.**
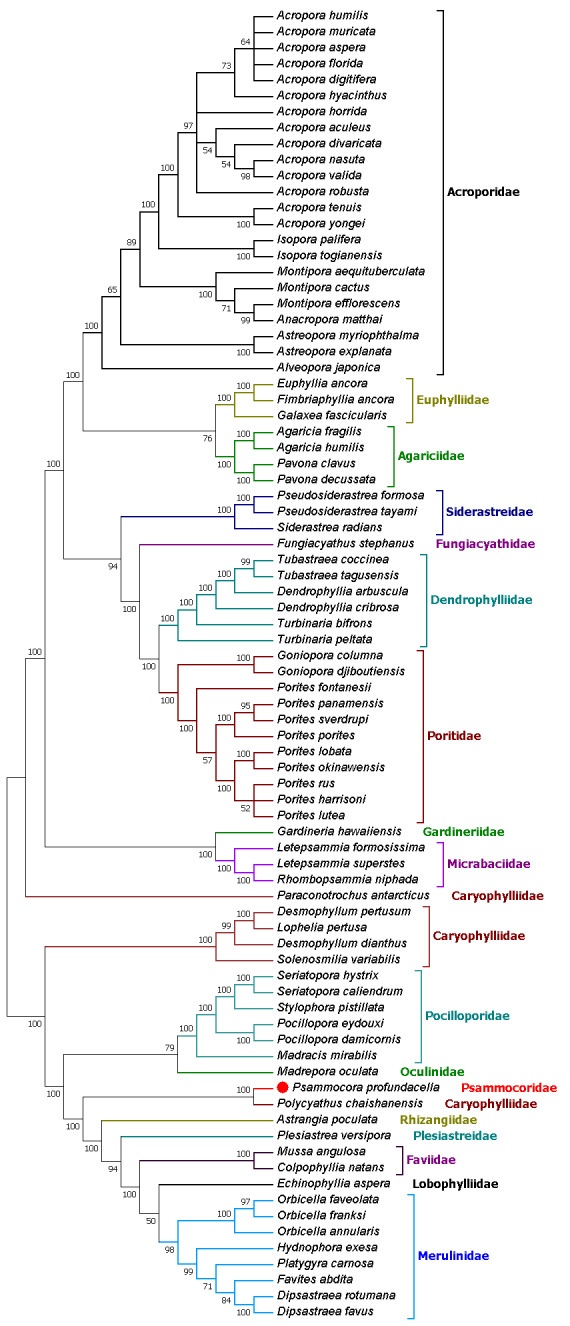
Inferred phylogenetic relationships, based on the concatenated nucleotide sequences of 13 mitochondrial PCGs, using Maximum Likelihood (ML) analysis. Numbers on branches are bootstrap percentages.

The ML tree topology of the 82 stony corals species showed that *P. profundacella* belongs to the “Robust” clade and is closely related to *Polycyathus chaishanensis* (Caryophylliidae) with high bootstrap support (Fig. 7). These findings are consistent with the results of Lin et al. (2012), which clearly showed that *Polycyathus chaishanensis* is closely related to a clade comprising *Psammocora*, *Coscinaraea*, *Leptastrea* and Fungiidae. The mitochondrial genome data have provided important molecular information for understanding evolutionary relationships amongst stony corals, but more variable markers and additional species should be sequenced to confirm the evolutionary relationships of Scleractinia in the future.

## Conclusions

The complete mitochondrial genome of *P. profundacella* was sequenced for the first time and it was also the first species in the family Psammocoridae whose mitogenome had been sequenced. The mitogenome of *P. profundacella* is 16,274 bp in size and shows similar gene order and gene composition with other scleractinian mitogenomes. The phylogenetic analysis of *P. profundacella*, on the basis of its mitochondrial protein-coding genes along with 81 other scleractinians, while preliminary, will help facilitate further studies on stony coral evolutionary and phylogenetic relationships.

## Supplementary Material

31D3D0DE-3760-52F1-A24D-795F78081DFE 10.3897/BDJ.9.e62395.suppl1 Supplementary material 1 Representative Scleractinia species included in this study for comparison Data type genomic File: oo_524724.pdf https://binary.pensoft.net/file/524724 Peng Tian

## Figures and Tables

**Table 1.** Organisation of the mitochondrial genome of *P. profundacella*.

**Gene**	**Position**		**Length (bp)**	**Anticodon**	**Codon**		Intergenic nucleotides*	**Strand**
	**From**	**To**			**Start**	**Stop**		
tRNA^Met^	1	72	72	CAU			1064	H
16S rRNA	233	1936	1704				160	H
ND5 5'	1970	2680	711		ATG		33	H
ND1	2813	3760	948		ATG	TAA	132	H
Cyt *b*	3768	4901	1134		TTA	TAA	7	H
ND2	5110	6213	1104		ATT	TAA	208	H
ND6	6214	6774	561		ATG	TAA	0	H
ATP6	6774	7451	678		ATG	TAA	-1	H
ND4	7451	8890	1440		ATG	TAG	-1	H
12S rRNA	9000	9905	906				109	H
COIII	9906	10685	780		ATG	TAA	0	H
COII	10842	11549	708		ATG	TAG	156	H
ND4L	11564	11830	267		TTG	TAA	14	H
ND3	11833	12174	342		ATG	TAA	2	H
ND5 3'	12230	13330	1101			TAG	55	H
tRNA^Trp^	13329	13398	70	UCA			-2	H
ATP8	13421	13615	195		ATG	TAA	22	H
COI	13669	15210	1542		ATG	TAA	53	H

**Notes**: * Data are numbers of nucleotides between the given gene and its previous gene, negative numbers indicate overlapping nucleotides; H indicated that the genes are transcribed on the heavy strand.

**Table 2.** Nucleotide composition in different regions of mitochondrial genome of *P. profundacella*.

**Gene/Region**	**T(%)**	**C(%)**	**A(%)**	**G(%)**	**A+T(%)**	**Size** (**bp)**
ND5	47.46	10.65	23.84	18.05	71.30	1812
ND1	45.89	11.18	23.21	19.73	69.10	948
Cyt *b*	48.94	10.67	23.99	16.40	72.93	1134
ND2	50.36	10.24	23.19	16.21	73.55	1104
ND6	49.73	10.70	23.89	15.69	73.62	561
ATP6	48.82	11.36	25.07	14.75	73.89	678
ND4	48.19	11.11	22.36	18.33	70.55	1440
COIII	44.74	13.21	22.31	19.74	67.05	780
COII	40.96	11.44	25.00	22.60	65.96	708
ND4L	43.82	10.49	25.09	20.60	68.91	267
ND3	50.00	9.36	19.01	21.64	69.01	342
ATP8	48.72	8.72	29.74	12.82	78.46	195
COI	42.41	13.16	23.80	20.62	66.21	1542
PCGs	46.80	11.20	23.60	18.40	70.40	11511
1**^st^**	37.00	12.60	23.20	27.00	60.20	3837
2**^st^**	48.19	17.96	18.37	15.48	66.56	3837
3**^st^**	55.07	3.15	29.11	12.67	84.18	3837
tRNA	26.06	19.01	30.28	24.65	56.34	142
rRNA	33.98	10.15	37.32	18.54	71.30	2610
Overall	43.17	11.32	26.34	19.17	69.51	16274
