# Supplementary material for: Complete mitochondrial DNA sequence of the Psammocora profundacella (Scleractinia, Psammocoridae): mitogenome characterisation and phylogenetic implications
Source: Biodivers Data J. 2021 Apr 19;9:e62395. doi: 10.3897/BDJ.9.e62395 (PMC8076163; doi:10.3897/BDJ.9.e62395)
Supplement: Supplementary material 1 — Representative Scleractinia species included in this study for comparison [file bdj-09-e62395-s001.pdf]

	Species	Family	Length (bp)	GenBank accession number
1	*Acropora aculeus*	Acroporidae	18,528	NC_029251
2	*Acropora digitifera*	Acroporidae	18,479	NC_022830
3	*Acropora divaricata*	Acroporidae	18,481	NC_022832
4	*Acropora florida*	Acroporidae	18,365	KF448533
5	*Acropora horrida*	Acroporidae	18,480	NC_022825
6	*Acropora hyacinthus*	Acroporidae	18,566	NC_022826
7	*Acropora muricata*	Acroporidae	18,481	NC_022824
8	*Acropora nasuta*	Acroporidae	18,481	NC_022831
9	*Acropora robusta*	Acroporidae	18,480	NC_022833
10	*Acropora tenuis*	Acroporidae	18,338	NC_003522
11	*Acropora yongei*	Acroporidae	18,342	NC_022829
12	*Astreopora myriophthalma*	Acroporidae	18,106	NC_024092
13	*Montipora aequituberculata*	Acroporidae	17,886	NC_037359
14	*Montipora efflorescens*	Acroporidae	17,886	NC_040137
15	*Acropora aspera*	Acroporidae	18,479	KF448532
16	*Acropora humilis*	Acroporidae	18,479	KF448528
17	*Acropora valida*	Acroporidae	18,385	MH141598
18	*Alveopora japonica*	Acroporidae	18,144	MG851913
19	*Anacropora matthai*	Acroporidae	17,888	NC_006898
20	*Astreopora explanata*	Acroporidae	18,106	KJ634269
21	*Isopora palifera*	Acroporidae	18,725	KJ634270
22	*Isopora togianensis*	Acroporidae	18,637	KJ634268
23	*Montipora cactus*	Acroporidae	17,887	NC_006902
24	*Agaricia fragilis*	Agariciidae	18,667	KM051016
25	*Agaricia humilis*	Agariciidae	18,735	NC_008160
26	*Pavona clavus*	Agariciidae	18,315	NC_008165
27	*Pavona decussata*	Agariciidae	18,378	KP231535
28	*Desmophyllum dianthus*	Caryophylliidae	16,310	KX000893
29	*Desmophyllum pertusum*	Caryophylliidae	16,149	KC875348
30	*Lophelia pertusa*	Caryophylliidae	16,150	FR821799
31	*Paraconotrochus antarcticus*	Caryophylliidae	17,562	MT409109
32	*Polycyathus chaishanensis*	Caryophylliidae	15,357	JF825140
33	*Solenosmilia variabilis*	Caryophylliidae	15,968	KM609293
34	*Dendrophyllia arbuscula*	Dendrophylliidae	19,069	KR824937
35	*Dendrophyllia cribrosa*	Dendrophylliidae	19,072	JQ290080
36	*Tubastraea coccinea*	Dendrophylliidae	19,094	KX024566
37	*Tubastraea tagusensis*	Dendrophylliidae	19,094	KX024567
38	*Turbinaria bifrons*	Dendrophylliidae	18,880	MT806020
39	*Turbinaria peltata*	Dendrophylliidae	18,966	NC_024671
40	*Euphyllia ancora*	Euphylliidae	18,875	JF825139
41	*Fimbriaphyllia ancora*	Euphylliidae	18,875	NC_015641
42	*Galaxea fascicularis*	Euphylliidae	18,751	NC_029696
43	*Colpophyllia natans*	Faviidae	16,906	NC_008162
44	*Mussa angulosa*	Faviidae	17,245	DQ643834
45	*Fungiacyathus stephanus*	Fungiacyathidae	19,381	JF825138
46	*Gardineria hawaiiensis*	Gardineriidae	19,430	MT376619
47	*Echinophyllia aspera*	Lobophylliidae	17,697	MG792550
48	*Dipsastraea favus*	Merulinidae	17,054	NC_046690

49	*Dipsastraea rotumana*	Merulinidae	16,466	MH119077
50	*Hydnophora exesa*	Merulinidae	17,790	MH086217
51	*Orbicella faveolata*	Merulinidae	16,138	AP008978
52	*Orbicella franksi*	Merulinidae	16,138	AP008975
53	*Platygyra carnosa*	Merulinidae	16,463	JX911333
54	*Favites abdita*	Merulinidae	17,825	NC_035879
55	*Orbicella annularis*	Merulinidae	16,138	NC_007224
56	*Letepsammia formosissima*	Micrabaciidae	19,048	MT705247
57	*Letepsammia superstes*	Micrabaciidae	19,073	MT706035
58	*Rhombopsammia niphada*	Micrabaciidae	19,542	MT706034
59	*Madrepora oculata*	Oculinidae	15,841	JX236041
60	*Plesiastrea versipora*	Plesiastreidae	15,320	MH025639
61	*Pocillopora damicornis*	Pocilloporidae	17,425	EU400213
62	*Pocillopora eydouxi*	Pocilloporidae	17,422	EF526303
63	*Seriatopora hystrix*	Pocilloporidae	17,059	EF633600.2
64	*Madracis mirabilis*	Pocilloporidae	16,951	NC_011160
65	*Seriatopora caliendrum*	Pocilloporidae	17,010	NC_010245
66	*Stylophora pistillata*	Pocilloporidae	17,177	NC_011162
67	*Goniopora columna*	Poritidae	18,766	JF825141
68	*Goniopora djiboutiensis*	Poritidae	18,765	NC_045931
69	*Porites fontanesii*	Poritidae	18,658	NC_037434
70	*Porites harrisoni*	Poritidae	18,630	NC_037435
71	*Porites lobata*	Poritidae	18,647	KU572435
72	*Porites lutea*	Poritidae	18,646	KU159432
73	*Porites okinawensis*	Poritidae	18,647	JF825142
74	*Porites panamensis*	Poritidae	18,628	KJ546638
75	*Porites porites*	Poritidae	18,648	DQ643837
76	*Porites rus*	Poritidae	18,647	NC_027526
77	*Porites sverdrupi*	Poritidae	18,628	KU956960
78	***Psammocora profundacella***	**Psammocoridae**	16,274	MT576637
79	*Astrangia poculata*	Rhizangiidae	14,853	NC_008161
80	*Pseudosiderastrea formosa*	Siderastreidae	19,475	KP260632
81	*Pseudosiderastrea tayami*	Siderastreidae	19,475	KP260633
82	*Siderastrea radians*	Siderastreidae	19,387	NC_008167
